# Dissipated energy inside the respiratory system during mechanical ventilation

**DOI:** 10.1186/cc13474

**Published:** 2014-03-17

**Authors:** M Gotti, C Chiurazzi, M Amini, C Rovati, M Brioni, G Rossignoli, A Cammaroto, C Bacile di Castiglione, S Luoni, K Nikolla, C Montaruli, T Langer, G Conte, M Cressoni, L Gattinoni

**Affiliations:** 1 Universita degli studi di Milano, Milan, Italy

## Introduction

During mechanical ventilation some of the energy delivered to the respiratory system (RS) is dissipated within it, while some is recovered during expiration. The amount of unrecovered energy represents mechanical work done on the RS by the ventilator and may be related to the development of ventilatory-induced lung injury (VlLI). The unrecovered energy is measured as the hysteresis area of the pressure-volume (PV) curve in static and dynamic conditions. We explored how and where the energy is dissipated inside the RS.

## Methods

In five piglets (weight 21 ± 2 kg) under general anesthesia, we recorded PV curves to quantify dynamic dissipated energy (DE) at increasing tidal volume (Tv) (150, 300, 450, 600, 750, 900) and at increasing respiratory rate (RR) (3, 6, 9, 12, 15). We then recorded PV curves for the same TV inflated with a super-syringe (100 ml), to quantify static DE. We also quantified airway DE connecting the postmortem isolated tracheobronchial tree to the ventilator and recording PV curves at the respiratory setting previously described.

## Results

**Figure 1:**
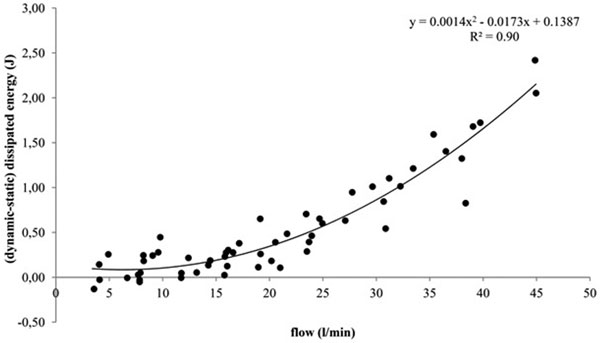
**Relationship between flow (l/minute) and (dynamic-static) dissipated energy (J)**.

Static DE(J) had a nonlinear relationship with the TV (ml/kg) applied: static DE = 0.0031*TV^15198^, *r*^2 ^= 0.96. Subtracting from the PV curve hysteresis area of a single breath (dynamic DE) the airway DE, the resulting curve overlapped static DE at every RR considered, suggesting that the amount of energy spent on the RS is equal to the static DE. Static DE can be estimated knowing flow (l/minute) and dynamic DE(J) since:(dynamic-static)DE = 0.0014*[flow]^2 ^- 0.0173*flow + 0.1387, *r*^2 ^= 0.90 (Figure 1).

## Conclusion

According to our data, the amount of energy that may be related to the development of VILI is static DE; it is a nonlinear function of TV and can be estimated knowing flow and dynamic DE.

